# Update of the results of a multinational survey regarding diagnosis and treatment of the temporomandibulare joint involvement in juvenile idiopathic arthritis- reflection of the day to day practice

**DOI:** 10.1186/1546-0096-9-S1-P110

**Published:** 2011-09-14

**Authors:** Ivan Foeldvari, Nikolay Tzaribachev, Angela Wierk, Randy Cron

**Affiliations:** 1 Hamburger Zentrum für Kinder- und Jugendrheumatologie, Hamburg, Germany; 2 Rheumazentrum, Bad Brahmstedt, Germany; 3 Pediatric Rheumatology, University of Birmingham, Alabama, USA

## Introduction

Temporomandibular joint (TMJ) involvement occurs up to 80% of patients with juvenile idiopathic arthritis (JIA). Currently they are no standardized procedures regarding diagnosis and treatment of this common presentation of JIA.

## Aim of the study

To assess the current clinical practices regarding diagnosis and treatment of TMJ involvement in JIA.

## Methods

Paediatric rheumatology colleagues were asked to fill out a survey with 8 items regarding diagnosis and treatment of TMJ involvement. The survey was distributed over the worldwide Paediatric Rheumatology electronic list-serve.

## Results

87 centres responded to the survey by Oktober 2010. Fortythree of the centres followed more than 300 patients with JIA. All responding centres were actively screening for TMJ involvement, 85 by history, all by physical exam and 2 by imaging. Seventy-seven (88%) were screening at first visit and 76 (87%) at each follow-up visit. If imaging was requested, 77% asked for MRI, 10% for ultrasound, 9% for CT and 33% for Xray. The centres reported the following prevalence of TMJ involvement: over 50% - 3 % of the centres, between 25 and 50% -10% of the centres; between 10% and 25% - 50% of the centres, less than 10% - 32% of the centres. The first line treatment of the TMJ involvement was a DMARD in 47%, an NSAID in 43%, an intraarticular corticosteroid injection in 34% and an anti-TNF agent in 6%. Overall, 57 of the centres (65%) were using intraarticular corticosteroid injections as treatment; of these centres 32 (56%) were using imaging. 20 centres gave details about imaging. MRI were used in 10% , CT in 30%, ultrasound in 45% and fluoroscopy in 15% as imaging for guidance during injections.

## Conclusion

TMJ arthritis is common among children with JIA, but a wide array of diagnostic and therapeutic approaches are being employed. An expert opinion/consensus statement regarding TMJ arthritis in JIA will likely benefit patients worldwide.

